# DAMPs, MAMPs, and NAMPs in plant innate immunity

**DOI:** 10.1186/s12870-016-0921-2

**Published:** 2016-10-26

**Authors:** Hyong Woo Choi, Daniel F. Klessig

**Affiliations:** Boyce Thompson Institute, Cornell University, 533 Tower Road, Ithaca, NY 14853 USA

**Keywords:** DAMPs, PAMPs, MAMPs, NAMPs, Innate immunity, Defense, Salicylic acid, Receptors

## Abstract

**Background:**

Multicellular organisms have evolved systems/mechanisms to detect various forms of danger, including attack by microbial pathogens and a variety of pests, as well as tissue and cellular damage. Detection via cell-surface receptors activates an ancient and evolutionarily conserved innate immune system.

**Result:**

Potentially harmful microorganisms are recognized by the presence of molecules or parts of molecules that have structures or chemical patterns unique to microbes and thus are perceived as non-self/foreign. They are referred to as Microbe-Associated Molecular Patterns (MAMPs). Recently, a class of small molecules that is made only by nematodes, and that functions as pheromones in these organisms, was shown to be recognized by a wide range of plants. In the presence of these molecules, termed Nematode-Associated Molecular Patterns (NAMPs), plants activate innate immune responses and display enhanced resistance to a broad spectrum of microbial and nematode pathogens. In addition to pathogen attack, the relocation of various endogenous molecules or parts of molecules, generally to the extracellular milieu, as a result of tissue or cellular damage is perceived as a danger signal, and it leads to the induction of innate immune responses. These relocated endogenous inducers are called Damage-Associated Molecular Patterns (DAMPs).

**Conclusions:**

This mini-review is focused on plant DAMPs, including the recently discovered Arabidopsis HMGB3, which is the counterpart of the prototypic animal DAMP HMGB1. The plant DAMPs will be presented in the context of plant MAMPs and NAMPs, as well as animal DAMPs.

## Background

All living organisms have evolved ways to protect themselves against abiotic and biotic assaults. For example, microbes utilize DNA restriction/modification systems to protect against foreign DNA; they also contain systems to detoxify and/or extrude xenobiotics or excessive reactive oxygen species (ROS). Multicellular organisms use other systems, and participation of one or more levels of immunity is often involved. The best studied and most appreciated in jawed vertebrates is the acquired/adaptive immune system with its well-known B and T cells and antigen-specific antibodies. This level of immunity is super-imposed on the much more fundamental, evolutionarily-ancient innate immune system, which is present not just in mammals but also in other animals and in plants. Only in the last several decades has the importance of innate immunity for the survival of multicellular organisms begun to be appreciated. It protects humans, other animals, and plants from the thousands of potentially-harmful microbes encountered daily. The development of innate immunity in multicellular organisms required the evolution of cell surface receptors that could recognize/bind molecules whose chemical structure/pattern is generally conserved within various classes of foreign organisms but is absent in “self” molecules. These conserved foreign (non-self) molecules are termed Microbe-Associated Molecular Patterns (MAMPs), also referred to as Pathogen-Associated Molecular Patterns (PAMPs), and their presence is detected by members of a large family of pattern recognition receptors (PRRs). PRRs activate one or more signaling pathways, often with the aid of co-receptors, to induce downstream defense responses. Examples of MAMPs include bacterial lipopolysaccharide, flagellin, EF-Tu, DNA, lipoproteins, peptidoglycans, and fungal chitin. Several excellent reviews of MAMPs are available [1–4].

In addition to biotic assault, organisms must cope with a variety of abiotic assaults such as mechanical or cellular damage, as well as environmental stresses like drought and salinity. Some endogenous molecules activate the innate immune system when they are released into the extracellular space (including plant apoplast) from their normal location due to damage (trauma); these molecules are referred to as Damage-Associated Molecular Patterns (DAMPs [3, 5]). DAMPs are passively released from dying cells due to damage, trauma, ischemia, or infection-induced necrosis. In addition, they can be actively secreted by certain immune cells or severely stressed cells (e.g. certain cancer cells [3]). While MAMPs are derived from microorganisms and activate the innate immune system, DAMPs are host cell derived and both initiate and perpetuate innate immune responses. It is generally accepted that these defenses help protect the damaged tissue, which is vulnerable to infection due to the disruption of physical barriers that would otherwise prevent microbial ingress. In mammals, inflammation is another component of the innate immune response; it not only helps to prevent/suppress infection, but also aids in healing.

This review will focus on DAMPs, particularly those of plants. DAMPs will be compared to MAMPs and to a newly-identified class of innate immunity activators termed Nematode-Associated Molecular Patterns (NAMPs [6]) since all three classes induce many of the same defense responses and share some signal transduction components.

### Animal DAMPs

We begin our discussion with animal DAMPs since they were first recognized and most extensively studied. The term DAMPs was coined by Seong and Matzinger in 2004 [7]. Table 1 lists 26 DAMPs, including purines, pyrimidines, DNA (unmethylated CpG), oxidized low-density lipoproteins, N-formyl peptides, and a variety of proteins. Cognate receptors for most have been identified (Table 1). In addition, some DAMPs form complexes with partner molecules/interactors to enhance or facilitate signaling. Among these is High Mobility Group Box 1 (HMGB1), which is one of the first identified and best characterized DAMP. HMGB1 is a highly abundant, chromatin-associated protein that is present in all animal cells [8]. It consists of two basic DNA-binding domains, designated HMG boxes A and B, and a highly acidic C-terminal tail that participates in specific intra-molecular interactions [9]. In the nucleus, HMGB1 binds the minor groove of DNA to facilitate DNA condensation, nucleosome formation, and transcription factor binding [10]. When it is released into the extracellular milieu from necrotic, damaged, or severely stressed cells, it functions as a DAMP with chemo-attractant and cytokine-inducing activities [11].

**Table 1:** Human DAMPs

DAMP		Receptor	Interactor	Reference
High Mobility Group Box 1 (HMGB1)		CXCR4 ^a^	CXCL12 ^b^	[14]
		TLR4 ^c^	CD14 ^d^/MD-2 ^e^	[15, 63]
		TLR4	LPS ^f^	[64, 65]
		TLR3/7/9	Nucleic acids	[66, 67]
		IL-1R1 ^g^	IL-1α/β ^h^	[68]
		TLR2	Nucleosome	[69]
		CD163 ^i^	Haptoglobin	[70]
		RAGE ^j^		[66, 71]
		Siglec-10 ^k^	CD24	[72]
		TIM3 ^l^		[73]
Heat Shock Protein (HSP)		TLR2/4	CD14	[74–77]
β-defensin		TLR4		[74, 78]
Peroxiredoxin-2 (PRDX2)				[79]
Calreticulin		CD91		[80, 81]
14-3-3η				[82–85]
Purines	Adenosine	P1		[86, 87]
	ADP	P2Y		[86–89]
	ATP	P2X/P2Y		[86, 87, 90–93]
Pyrimidines	UDP	P2Y		[86, 87, 94]
	UDP-glucose	P2Y		[86, 87, 95]
Amyloid β		TLR4/6	CD36	[74, 96]
		RAGE		[97–99]
		FPRL1 ^m^		[100]
		NLRP3 ^n^		[101]
S100/calgranulin		RAGE		[102]
		TLR4		[103]
Uric acid		TLR2/4	CD14	[104, 105]
		NLRP3		[106]
Degradation product of ECM ^o^	Biglycan	TLR2/4		[107, 108]
	Hyaluronan	TLR2/4	CD44/MD-2	[30, 109–111]
	Versican	TLR2/6	CD14/MD-2	[112]
	Extra-domain A of fibronectin	TLR4		[113]
	Surfactant protein A	TLR2		[114, 115]
Oxidized LDL ^p^		TLR4/6	CD36	[74, 116, 117]
		TLR4		[118]
		SR ^q^		[119]
Oxidized phospholipids		PPARα ^r^		[120, 121]
		TLR2/4	CD14/MD-2	[121–124]
mitochondrial DAMPs	DNA (unmethylated CpG)	TLR9		[125]
	ATP	P2X/P2Y		[86, 87, 90–93]
	TFAM ^s^			[126]
	N-formyl peptides	FPRs ^t^		[127]
	Succinate			[128]
	Cardiolipin	NLRP3		[129]

^a^ CXCR4: chemokine (C-X-C motif) receptor 4; ^b^ CXCL12: chemokine (C-X-C motif) ligand 12; ^c^ TLR: toll-like receptor; ^d^ CD: cell differentiation antigen; ^e^ MD-2: myeloid differentiation protein-2; ^f^ LPS: lipopolysaccharides; ^g^ IL-1R1: interleukin 1 receptor, type I; ^h^ IL: interleukin; ^i^ CD163: cluster of differentiation 163; ^j^ RAGE: receptor for advanced glycation end products; ^k^ Siglec-10: sialic acid binding Ig-like lectin -10; ^l^ TIM3: T-cell immunoglobulin mucin receptor 3; ^m^ FPRL1: formyl peptide receptor-like 1; ^n^ NLRP: NOD-like receptor protein; ^o^ ECM: extracellular matrix components; ^p^ LDL: low density lipoprotein; ^q^ SR: Scavenger Receptor; ^r^ PPARα: peroxisome proliferator-activated receptor alpha; ^s^ TFAM: mitochondrial transcription factor A; ^t^ FPRs: formyl peptide receptors

Extracellular HMGB1 mediates a range of biological responses in association with multiple receptors, such as the Receptor for Advanced Glycation End products (RAGE), Toll-like receptor 2 (TLR2), TLR4, TLR9, C-X-C chemokine receptor type 4 (CXCR4), Siglec-10, and T-Cell Immunoglobulin Mucin Receptor 3 (TIM3) [11, 12]. Notably, specific heterocomplex formation between HMGB1 and a variety of interactors, such as adaptor MD-2 or pro-inflammatory ligands lipopolysaccharides, and CpG oligodeoxynucleutides, enhances or facilitates signaling and in some cases is critical for HMGB1’s recognition by distinct receptors (Table 1). The specific heterocomplex formation appears to be at least partially regulated by the different redox states of HMGB1, which in part depend on a reversible intra-molecular disulfide bond formed between cysteine residues 23 and 45 [12, 13]. Recent studies showed that reduced HMGB1 forms a heterocomplex with CXCL12, which promotes the recruitment of inflammatory cells to damaged tissue through recognition by the CXCR4 receptor [14]. Disulfide bond-containing HMGB1 specifically binds MD-2, which facilitates recognition by TLR4, leading to induction of the NF-κB-mediated transcriptional activation of pro-inflammatory cytokines [13, 15]. HMGB1 also interacts with several other receptors, including RAGE and TLR2; it is presently unclear whether specific redox states are required for its recognition by these receptors [11]. HMGB1’s diverse activities, partner molecules, and receptors likely account for its multiple roles in many prevalent, devastating human diseases.

We recently discovered that HMGB1 binds salicylic acid (SA); this suppresses both reduced HMGB1’s chemo-attractant activity and disulfide bond-containing HMGB1’s ability to induce the expression of pro-inflammatory cytokine genes and COX-2 [16]. The SA-binding sites on HMGB1 were identified in the HMG-box domains by NMR studies and confirmed by mutational analysis. A HMGB1 protein mutated in one of the SA-binding sites retained chemo-attractant activity, but lost binding of and inhibition by SA, thereby firmly establishing that SA binding to HMGB1 directly suppresses its pro-inflammatory activities. Natural and synthetic SA derivatives with much greater potency for inhibition of HMGB1 also were identified, thereby providing proof-of-concept that new SA-based molecules with high efficacy are achievable.

### Plant DAMPs

In contrast to animals, many fewer DAMPs have been identified in plants to date (Table 2). The largest and arguably the best-characterized class are polypeptides/peptides produced from larger precursor proteins. These include three families discovered by Ryan and his colleagues during their studies to identify systemin – a term “used to describe polypeptide defense signals that are produced by the plant in response to physical damage and that induce defense genes, either locally or systemically” [17]. An 18 amino acid (aa) polypeptide was isolated from 60 lb of tomato seedling and shown to induce the synthesis of wound-inducible proteinase inhibitor proteins [18]. This tomato systemin is generated by wound-induced processing of a 200 aa prohormone prosystemin, which is located in the cytoplasm of vascular phloem parenchyma cells. Systemin induces the neighboring companion cells and sieve elements of the vascular bundle to synthesize jasmonic acid (JA), which in turn systemically activates the expression of proteinase inhibitor genes [19–21].

**Table 2:** Plant DAMPs

DAMP	Receptor	Co-receptor	Reference
Systemin	SR160 ^a^	n.d.	[18, 22]
Hydroxyproline-rich systemin	n.d.	n.d.	[130–134]
Plant elicitor peptides (Peps)	PEPR1/2 ^b^	BAK1 ^c^ and BKK1 ^d^	[17, 18, 23, 25, 27, 28, 135, 136]
Oligogalacturonides (OGs)	WAK1 ^e^	n.d.	[31, 33, 34, 137, 138]
Extracellular ATP (eATP)	DORN1 ^f^	n.d.	[37, 38]
*At*HMGB3 ^g^	n.d.	BAK1 and BKK1	[40]

*n.d.* not determined

^a^ SR160: 160-kDa systemin cell-surface receptor; ^b^ PEPR: PEP receptor; ^c^ BAK1: BRI1-Associated receptor Kinase 1; ^d^ BKK1: BAK1-LIKE Kinase 1; ^e^ WAK1: Wall-Associated Kinase 1; ^f^ DORN1: Does Not Respond to Nucleotides 1; ^g^ *At*HMGB3: *Arabidopsis thaliana* High Mobility Group Box 3 protein

While systemin is present in many other Solanaceous species, including potato, pepper and nightshade [22], it is not found in tobacco. This finding prompted Ryan’s group to search for another type of systemin. Ultimately, two hydroxyproline-rich 18 aa polypeptides, that are processed from a 165 aa preproprotein but share no sequence homology with the tomato systemin, were identified [17].

A third family of peptide-based DAMPs was discovered in Arabidopsis [23]. These 23 aa plant elicitor peptides (Peps) are derived from a 92 aa precursor. Two receptors have been identified for *At*Pepl, PEPR1, and PEPR2 [24, 25]. *At*Peps induce a variety of innate immune responses and enhanced resistance, and a form of precursor ProPep3 was recently shown to be released into the extracellular space upon infection of Arabidopsis with hemi-biotrophic *Pseudomonas syringae* [26]. A maize (*Zea mays*) ortholog, *Zm*Pep1, was subsequently identified and shown to enhance resistance to microbial pathogens, just like *At*Pepl [27]. For a more in-depth discussion of endogenous peptide elicitors, see Yamaguchi and Huffaker [28].

Another class of DAMPs found in plants, as well as animals, is derived from the extracellular matrix. In vertebrates fragments of hyaluronan, a simple linear polysaccharide consisting of repeating D-glucuronic acid and D-N-acetylglucosamine, induce innate immunity when released by mechanical damage or hydrolytic enzymes [29]. These fragments are perceived by the leucine-rich repeat-containing TLR2 and TLR4 receptors [29, 30]. Similarly, plants contain the pectic polysaccharide homogalacturonan, a linear polymer of 1, 4-linked α-D galacturonic acid, which helps maintain cell wall integrity. Fragments of this polymer, called oligogalacturonides (OGs), can be released mechanically or more commonly by pathogen-encoded hydrolytic enzymes. OGs induce innate immune responses, including MAPK activation, callose deposition, ROS production, elevated cytosolic Ca^2+^, and defense gene activation [31, 32]. The wall-associated kinase 1 (WAK1) has been identified as a likely receptor for OGs [33, 34].

Extracellular ATP (eATP) comprises yet another class of plant DAMPs found in both plants and animals. Despite decades of mounting evidence that eATP acts as a signaling molecule, this function was largely discounted/discredited, probably because of ATP’s ubiquitous nature and central role as the universal energy currency in all living organisms from bacteria to humans [35, 36]. Only with the identification of its plasma membrane-localized receptors, first in animals (see [35]) and then in plants [37], was its signaling function accepted in both kingdoms. In animals eATP acts as a neurotransmitter and signaling molecule that participates in muscle contraction, cell death, and inflammation [35]. Two types of receptors are involved: a G protein-coupled P2Y receptor and a ligand-gated ion channel P2X receptor. In plants eATP’s signaling role was more recently confirmed with the identification of its receptor, Does not Respond to Nucleotides 1 (DORN1 [37]). eATP’s designation as a plant DAMP is based on the combined observations that i) the *dorn1* mutant displays suppressed transcriptional response not only to ATP but also to wounding, ii) most of the genes induced by application of eATP are also wound-inducible [36], and iii) eATP treatment induces typical innate immune responses, including cytosolic Ca^2+^ influx, MAPK activation, and induction of dense-associated genes, including some involved in the biosynthesis of JA and ethylene [36, 38, 39]. However, it is not yet known whether it contributes to resistance to pathogens.

We recently identified a fourth class of plant DAMPs, the Arabidopsis HMGB protein *At*HMGB3 [40]. All eukaryotic cells, including plants, have HMGB1-related proteins. In Arabidopsis, 15 genes encode HMG-box domain-containing proteins. They have been subdivided into four groups: (i) HMGB-type proteins, (ii) A/T-rich interaction domain (ARID)-HMG proteins, (iii) 3xHMG proteins that contain three HMG boxes, and (iv) the structure-specific recognition protein 1 (SSRP1) [41]. Based on their nuclear location and domain structure, the eight HMGB-type proteins (HMGB1/2/3/4/5/6/12/14) are thought to function as architectural chromosomal proteins, similar to mammalian HMGB1. Notably, *At*HMGB2/3/4 are present in the cytoplasm and as well as the nucleus [41–43]. The cytoplasmic function of these proteins is not known. However, the cytoplasmic subpopulations should have greater access to the extracellular space (apoplast) after cellular damage as compared to the *At*HMGBs located exclusively in the nucleus [41–43], since they are not bound to DNA and need only cross the plasma membrane to enter the apoplast. Given the well-established role of mammalian HMGB1 as the prototypic DAMP, the presence of a cytoplasmic subpopulation of *At*HMGB3 raised the possibility that this protein serves a similar function. Indeed, when recombinant *At*HMGB3 was infiltrated into Arabidopsis leaves, it exhibited DAMP-like activities similar to those of *At*Pep1. Treatment with either protein induced MAPK activation, callose deposition, defense-related gene expression, and enhanced resistance to necrotrophic *Botrytis cinerea* [40].

In contrast to mammalian HMGB1, which can be actively secreted following post-translational modification, there is no evidence for secretion of *At*HMGB3. It probably enters the extracellular space passively when cells are damaged mechanically, such as by insects, or during infection by necrotrophic pathogens. Indeed *B. cinerea* infection caused release of *At*HMGB3 into the apoplast within 24 h after inoculation. Such rapid release during the early phase of cellular necrosis induced by necrotrophs could enhance resistance by activating immune responses [40].

Additional analyses revealed that *At*HMGB3, like HMGB1, binds SA, and that this interaction, which is mediated by conserved Arg and Lys residues in *At*HMGB3’s single HMG box, inhibits its DAMP activity [40]. This finding appears to conflict with SA’s well-known role as a positive regulator of immune responses [44–47]. However, while SA-induced defense responses are critical for resistance to biotrophic and hemi-biotrophic pathogens, the main hormone responsible for activating defenses against necrotrophic pathogens and insects is JA [44, 45]. The JA and SA defense signaling pathways are generally mutually antagonistic [48]. SA-mediated inhibition of *At*HMGB3’s DAMP activity may therefore provide one mechanism through which these pathways crosstalk. In this scenario, cellular damage caused by infection with necrotrophic pathogens would lead to the release of *At*HMGB3 into the extracellular spaces; this would activate JA/ethylene-associated defenses to help neutralize this threat. In contrast, infection by biotrophic pathogens induces SA biosynthesis [44, 45]. Increased SA levels could then antagonize the activation of JA-associated defenses by suppressing *At*HMGB3’s DAMP activity, as well as promote the activation of SA-associated defenses that are more effective against this type of pathogen [40].

The discovery that extracellular *At*HMGB3 is a plant DAMP whose immune response-inducing activity is inhibited by SA binding provides cross-kingdom evidence that HMGB proteins function extracellularly as DAMPs in both plants and animals. Moreover, it highlights the existence of common targets and shared mechanisms of action for SA in plants and humans. Interestingly, the majority of plant DAMPs identified to date have counterparts in animals. Our studies have further indicated that plants and animals share common targets of SA beyond the HMGBs [46]. For example, the glycolytic enzyme glyceraldehyde 3-phosphate dehydrogenase (GAPDH) in both plants and humans binds SA and as a result has altered activity. SA suppresses GAPDH’s roles in replication of Tomato Bushy Stunt Virus in plants and may have similar effects on hepatitis C virus replication in humans [49]. It also suppresses GAPDH-mediated neuronal cell death in animals [50]. Preliminary analyses of high-throughput screens suggest the existence of many more SA targets in both plants and humans. Perhaps the presence of multiple SA targets in animals evolved in response to either ingestion of low levels of SA that are naturally present in plant material, or endogenous synthesis of SA from benzoates [46]. Future studies will be required to assess whether these novel plant and animal SA-interacting proteins function as DAMPs.

### NAMPs

Nematodes, one of the most abundant animals in nature, parasitize both plants and animals. Several studies indicated that plants could perceive infection by nematodes [51–53], but the identity of the perceived nematode-derived signal was unknown. We recently identified a group of defense signaling molecules from several genera of plant-parasitic nematodes, including both root-knot and cyst nematodes [6]. They are an evolutionarily conserved family of nematode pheromones called ascarosides. Ascr#18, the most abundant ascaroside in plant-parasitic nematodes, induces hallmark innate immune responses including activation of i) MAPKs, ii) defense genes, and iii) the SA and JA defense-signaling pathways, as well as, enhanced resistance to viral, bacterial, fungal, and oomycete pathogens and root-knot nematodes in several dicot and monocot plant species.

### MAMPs, DAMPs, and NAMPs

Although the sources of the inducing signals are very different, with MAMPs derived from microbes, NAMPs derived from nematodes, and DAMPs being aberrantly-located endogenous molecules, studies of Arabidopsis suggest that most members of these three classes of immune-inducing molecules activate innate immune signaling via pathways that share the same leucine-rich repeat receptor-like kinases BRI1-Associated Kinase1 (BAK1) and BAK1-Like Kinase1 (BKK1) ([1, 54–56], for NAMP unpublished result M. Manohar, F.C. Schroeder, and D.F. Klessig). In addition, these molecules induce many of the same innate immune defense responses, including an influx of Ca^+2^ into the cytosol, callose deposition, activation of the defense-associated MAPKs MPK3 and MPK6, production of ROS, and enhanced expression of many defense-related genes (Table 3). Plant receptors have been identified for several MAMPs, such as FLS2 for flagellin/flg22 [57] and EFR for EF-Tu/elf18 [58]. Receptors for most of the plant DAMPs have also been discovered, including Arabidopsis PEPR1/2 for Peps [24, 25], Arabidopsis WAK1 for OGs [33, 59], and Arabidopsis DORN1 for eATP [37]. While tomato SR160 was initially reported as the receptor for systemin [60], two recent studies argue that it is not [61, 62]. The plant receptors for *At*HMGB3 and the ascaroside NAMP ascr#18 remain unknown (Table 2). Nor is it known whether *At*HMGB3’s DAMP signaling is enhanced or facilitated by interacting molecules as has been shown for mammalian HMGB1.

**Table 3:** Comparison of the innate immune responses and signaling components in Arabidopsis that are induced or utilized by MAMPs, NAMPs, and DAMPs

Inducers	Innate immune responses					Signaling components
	Ca^2+^ influx	Callose deposition	MPK3/MPK6 activation	ROS production	Defense gene expression	BAK1/BKK1
MAMPs^a^	√	√	√	√	√	√
NAMPs	n.d.	n.d.	√	√ ^b^	√	√ ^c^
DAMPs						
Systemin	n.d.	n.d.	n.d.	n.d.	√	n.d.
Hydroxyproline-rich systemin	n.d.	n.d.	n.d.	n.d.	√	n.d.
Plant elicitor peptides (Peps)	√	√	√	√	√	√
Oligogalacturonides (OGs)	√	√	√	√	√	√
Extracellular ATP (eATP)	√	n.d.	√	√	√	n.d.
*At*HMGB3 ^d^	n.d.	√	√	n.d.	√	√

√ = yes; n.d. = not determined

^a^ Note most/many, but not all, MAMPs have been shown to utilize the BAK1/BKK1 signaling pathway and induce these innate immune responses

^b^ Unpublished data – S. Hind, G.B. Martin, P. Manosalva, F.C. Schroeder, D.F. Klessig

^c^ Unpublished data – M. Manohar, F.C. Schroeder, D.F. Klessig

^d^ *Arabidopsis thaliana* High Mobility Group Box 3 protein

## Conclusions

Only during the past two decades has the importance of DAMPs for the survival of multicellular organisms emerged; this finding has fostered an active area of investigation. Compared to the more than two dozen DAMPs discovered in animals to date, relatively few have been identified in plants. Most of these plant DAMPs have counterparts in animals, including eATP, HMGBs, extracellular matrix fragments (e.g. OGs), and peptides processed from larger precursor proteins (e.g. systemin and Peps). Future investigations are likely to reveal many more shared DAMPs. Interestingly, DAMPs induce similar innate immune responses in plants as do microbe-derived MAMP and nematode-derived NAMPs. Furthermore, most DAMPs, MAMPs, and NAMPs appear to activate innate immune signaling via BAK1 and BKK1. This observation suggests that efforts to elucidate the pathway(s) through which innate immunity is activated will likely identify additional signaling components that are shared by these three classes of inducers.

## Abbreviations

Ascr: Ascaroside
BAK1: BRI1-Associated Kinase1
BKK1: BAK1-Like Kinase1
DAMP: Damage-associated molecular pattern
DORN1: Does not Respond to Nucleotides1
eATP: Extracellular adenosine triphosphate
EFR: Elongation factor Tu receptor
FLS2: Flagellin sensitive2
HMGB: High mobility group box protein
JA: Jasmonic acid
MAMP: Microbe-associated molecular pattern
MAPK: Mitogen-activated protein kinase
NAMP: Nematode-associated molecular pattern
NMR: Nuclear magnetic resonance
OG: Oligogalacturonides
Pep: Plant elicitor peptide
PEPR: Pep receptor
PRR: Pattern recognition receptor
ROS: Reactive oxygen species
SA: Salicylic acid
WAK1: Wall-associated kinase1
